# Familiar Letters on Chemistry, and Its Relation to Commerce, Physiology, and Agriculture

**Published:** 1844-01

**Authors:** 


### Art. XVI.

*Familiar Letters on Chemistry, and its relation to Commerce, Physiology, and Agriculture.* By Justus Liebig, m.d. ph.d. f.r.s., Professor of Chemistry in the University of Giessen. Edited by John Gardner, m.d., Member of the Chemical Society.—*London,* 1843. 12mo, pp. 180.

These letters, as we learn from the author's preface, were intended to be mere sketches of some of the most important subjects on which chemistry comes into immediate relation with human welfare; and were written for the especial purpose of exciting the attention of governments and an enlightened public, to the necessity of establishing schools of chemistry, and of promoting, by every means, the study of a science so intimately connected with the arts, pursuits, and social well-being, of modern civilized nations. In consequence of the publication of some of these letters in Germany, new professorships have been established in the universities of Göttingen and Würtzburg, for the express purpose of facilitating the application of chemical truths to the practical arts of life, and of following up the new line of investigation and research,—the bearing of chemistry upon physiology, medicine, and agriculture,—which may be said to be only just begun. "For my own part," says the author, "I do not scruple to avow the conviction, that ere long a knowledge of the principal truths of chemistry will be expected in every educated man; and that it will be as necessary to the statesman and political economist, and to the practical agriculturist, as it is already indispensable to the physician and the manufacturer."

The first five of these letters embrace several interesting facts relating to the application of chemistry to manufacturing processes,—such as the production of soda, sulphuric acid, soap, &c.; the remaining eleven contain a *resumé* of the author's doctrines, in regard to the bearing of chemistry upon animal and vegetable physiology, agriculture, &c.,—subjects which are more fully discussed in his larger treatises. His views are here presented in a very clear and definite form; and there is a studied avoidance of controverted questions, whilst great stress is laid upon a few prominent and easily-substantiated facts, which have an important practical bearing. Such is especially the case, in the portion which relates to agriculture; and we shall take this opportunity of directing the attention of our readers to the views at which Professor Liebig has arrived subsequently to our review of his 'Organic Chemistry applied to Agriculture;' referring to the third edition of that work, just published, for a more detailed enunciation of them, and for the data on which they are founded.

It will be remembered that many facts and arguments were adduced by Liebig in proof of the position that vegetables, during their ordinary growth, derive little or none of their carbon from the *humus* of the soil, but are indebted for it entirely to the atmosphere. The case of a plantation on a barren mountain side, which goes on year after year increasing in size, and at the same time adding to, rather than diminishing, the quantity of carbon in the soil, was especially adverted to. He has now adopted similar views respecting the source of the nitrogen, which the plants cultivated for food take up in large quantities; and the following circumstance appears to prove this beyond a doubt. The materials of cheese are entirely derived from the plants which serve as food for cows. The meadows of Holland have, in the lapse of centuries, produced millions of hundreds weight of cheese, of which a large proportion has been exported from the country, and of which, therefore, the azotized materials have been completely withdrawn, without any corresponding return being made. The only manure with which these meadows have been supplied, consists of the solid and fluid excrements of the cattle which pasture upon them; and these return to the soil the saline and earthy ingredients which have been withdrawn from it, but only a portion of the carbon and nitrogen that entered into the composition of the food. Yet these meadows retain their fertility, which could not be the case if more carbon and nitrogen were thus withdrawn than could be supplied by the atmosphere.

The great value of manures consists, therefore, according to Prof.

Liebig, in their imparting to the soil the *mineral* ingredients required by the particular kind of plants which are to be raised. It is true that, by the use of manures which yield a copious supply of carbonic acid and ammonia, *time* may be saved,—the growth of the plant being forced by the increased supply of food; but these will be totally inefficacious, if the mineral matters be not supplied in an equivalent proportion; and the latter used alone will frequently answer the required purpose. Now all those vegetable substances which are cultivated for the food of man and animals, from our experience of their nutritive properties, contain a considerable amount of the alkaline and earthy *phosphates;* and the maintenance of a sufficient supply of these in the soil should be, in Prof. Liebig's estimation, the great object of the agriculturist. We give large extracts from his closing letter, on account of the vast practical importance of the subject, and our desire to lose no opportunity of calling general attention to his suggestions.

"An enormous quantity of these substances, indispensable to the nourishment of plants, is annually withdrawn from the soil, and carried into great towns, in the shape of flour, cattle, &c. It is certain that this incessant removal of the phosphates must tend to exhaust the land, and diminish its capability of producing grain. The fields of Great Britain are in a state of progressive exhaustion from this cause, as is proved by the rapid extension of the cultivation of turnips and mangel-wurzel,—plants which contain the least amount of the phosphates, and therefore require the smallest quantity for their development. These roots contain 80 to 92 per cent. of water. Their great bulk makes the amount of produce fallacious as respects their adaptation to the food of animals; inasmuch as their contents of the ingredients of the blood, i. e. of substances which can be transformed into flesh, stands in a direct ratio to their amount of phosphates, without which neither blood nor flesh can be formed.

Our fields will become more and more deficient in these essential ingredients of food, in all localities where custom and habits do not admit the collection of the fluid and solid excrements of man, and their application to the purposes of agriculture. In a former letter I showed you how great a waste of phosphates is unavoidable in England, and referred to the well-known fact, that the importation of bones restored in a most admirable manner the fertility of the fields exhausted from this cause. In the year 1827 the importation of bones for manure amounted to 40,000 tons; and Huskisson estimated their value to be from £100,000 to £200,000 sterling. The importation is still greater at present; but it is far from being sufficient to supply the waste.

"Another proof of the efficacy of the phosphates in restoring fertility to exhausted land, is afforded by the use of the *guano,*—a manure which, although of recent introduction into England, has found such general and extensive application.

"We believe that the importation of one cwt. of *guano* is equivalent to the importation of eight cwt. of wheat; the cwt. of guano assumes, in a time which can be accurately estimated, the form of a quantity of food, corresponding to eight cwt. of wheat. The same estimate is applicable in the valuation of bones.

"If it were possible to restore to the soil of England and Scotland the phosphates which during the last fifty years have been carried to the sea by the Thames and the Clyde, it would be equivalent to manuring with millions of cwts. of bones, and the produce of the land would increase one third, or perhaps double itself, in five to ten years.

"We cannot doubt that the same result would follow if the price of the guano admitted the application of a quantity to the surface of the fields, containing as much of the phosphates as have been withdrawn from them in the same period.

"If a rich and cheap source of phosphate of lime and the alkaline phosphates were open to England, there can be no question that the importation of foreign corn might be altogether dispensed with in a short time. For these materials England is at present dependent upon foreign countries, and the high price of guano and of bones prevents their general application and in sufficient quantities. Every year the trade in these substances must decrease, or their price will rise as the demand for them increases.

"According to these premises, it cannot be disputed that the annual expense of Great Britain for the importation of bones and guano is equivalent to a duty on corn; with this difference only, that the amount is paid to foreigners in money.

"To restore the disturbed equilibrium of constitution of the soil,—to fertilize her fields,—England requires an enormous supply of animal excrements; and it must therefore excite considerable interest to learn, that she possesses beneath her soil beds of fossil *guano*,—strata of animal excrements, in a state which will probably allow of their being employed as a manure at a very *small* expense. The coprolithes, discovered by Dr. Buckland, (a discovery of the highest interest to geology,) are these excrements; and it seems extremely probable that in the strata England possesses the means of supplying the place of recent bones, and therefore the principal conditions of improving agriculture,—of restoring and exalting the fertility of her fields.

"In the autumn of 1842, Dr. Buckland pointed out to me a bed of coprolithes in the neighbourhood of Clifton, from half to one foot thick, inclosed in a limestone formation; extending, as a brown stripe in the rocks, for miles along the banks of the Severn. The limestone marl of Lyme-Regis consists for the most part of one fourth part of fossil excrements and bones. The same are abundant in the lias of Bath, Eastern and Broadway Hill. Near Eversham, Dr. Buckland mentions beds several miles in extent, the substance of which consists, in many places, of a fourth part of coprolithes.

"Pieces of the limestone rock of Clifton, near Bristol, which is rich in coprolithes, and organic remains, fragments of bones, teeth, &c., were subjected to analysis, and were found to contain above 18 per cent. of phosphate of lime. If this limestone is burned and brought in that state to the fields, it must be a perfect substitute for bones, the efficacy of which as a manure does not depend upon the nitrogenized matter, as has been generally but erroneously supposed, which they contain, but on their phosphate of lime.

"The osseous breccia found in many parts of England deserves especial attention, as it is highly probable that in a short time it will become an important article of commerce.

"What a curious and interesting subject for contemplation! In the remains of an extinct *animal* world England is to find the means of increasing her wealth in agricultural produce, as she has already found the great support of her manufacturing industry in fossil fuel,—the preserved matter of primeval forests, the remains of a *vegetable* world. May this expectation be realized; and may her excellent population be thus redeemed from poverty and misery." (pp. 174-9.)

We suspect that the learned Professor of Oxford must have unintentionally misled the Giessen chemist, by the enthusiasm of manner which he probably showed when demonstrating to him his favorite bed of coprolithes. For, on the information of a friend well versed in the geology of the places alluded to, we may state that it would be no easy matter to collect from them a cart-load of coprolithes; so that Britain must not place too much reliance on her *fossil* stores of phosphate of lime, but must devise means of taking care of the *recent*.

